# Characterization of gut microbiota dysbiosis of diarrheic adult yaks through 16S rRNA gene sequences

**DOI:** 10.3389/fvets.2022.946906

**Published:** 2022-09-09

**Authors:** Zhou-Lin Wu, Ranlei Wei, Xueqin Tan, Danjiao Yang, Dayu Liu, Jiamin Zhang, Wei Wang

**Affiliations:** ^1^Key Laboratory of Meat Processing of Sichuan, College of Food and Biological Engineering, Chengdu University, Chengdu, China; ^2^National Frontier Center of Disease Molecular Network, West China Hospital, Sichuan University, Chengdu, China; ^3^Institute of Animal Science of Ganzi Tibetan Autonomous Prefecture of Sichuan Province, Kangding, China

**Keywords:** yak, diarrhea, gut microbiota, dysbiosis, 16S rRNA

## Abstract

The ruminant gut microbial community has a strong impact on host health and can be altered during diarrhea disease. As an indigenous breed of the Tibetan Plateau, domestic yak displays a high diarrhea rate, but little research has been done to characterize the bacterial microbial structure in diarrheic yaks. In the present study, a total of 30 adult yaks, assigned to diarrhea (case, *N* = 15) and healthy (control, *N* = 15) groups, were subjected to gut microbiota profiling using the V3–V4 regions of the 16S rRNA gene. The results showed that the gut microbiome of the case group had a significant decrease in alpha diversity. Additionally, differences in beta diversity were consistently observed for the case and control groups, indicating that the microbial community structure was changed due to diarrhea. Bacterial taxonomic analysis indicated that the Bacteroidetes, Firmicutes, and Proteobacteria were the three most dominant phyla in both groups but different in relative abundance. Especially, the proportion of Proteobacteria in the case group was increased as compared with the control group, whereas Spirochaetota and Firmicutes were significantly decreased. At the genus level, the relative abundance of *Escherichia-Shigella* and *Prevotellaceae_UCG-003* were dramatically increased, whereas that of *Treponema, p-2534-18B5_gut_group*, and *Prevotellaceae_UCG-001* were observably decreased with the effect of diarrhea. Furthermore, based on our linear discriminant analysis (LDA) effect size (LEfSe) results, *Alistipes, Solibacillus, Bacteroides, Prevotellaceae_UCG_003*, and *Bacillus* were significantly enriched in the case group, while the other five genera, such as *Alloprevotella, RF39, Muribaculaceae, Treponema*, and *Enterococcus*, were the most preponderant in the control group. In conclusion, alterations in gut microbiota community composition were associated with yak diarrhea, differentially represented bacterial species enriched in case animals providing a theoretical basis for establishing a prevention and treatment system for yak diarrhea.

## Introduction

Qinghai-Tibetan plateau with an average altitude exceeding 4,000 m is known as the roof ridge of the world, where most animals cannot survive because of the harsh climate, hypoxia, and low atmospheric pressure (1). Yak (*Bos grunniens*), a multifunctional and dominant livestock species on the Qinghai-Tibetan plateau, has occurred in these regions ≈7,300 years before present (BP). In spite of the extremely hostile environment, yaks have well adapted to the Qinghai-Tibetan plateau and provided a stable source of food and labor for people indigenous to these regions (2). By contrast to other ruminants, yaks have a unique rumen microbial ecosystem that enables them to evolve special adaptations in physiology and nutrient metabolism (3).

In bovines, the rumen is described as a “black box” due to the multifarious microbes, and the gene content of microbes is hundreds of times that of host cells (4). Previous studies have shown that bovines possess trillions of gut microbes that include a variety of bacteria, protozoa, and fungi. Among them, fungi account for approximately 0.1%, whereas bacteria account for approximately 98% (5). These microbes display a broad range of symbiotic interactions with the host, some microbes degrade plant fibers, non-fiber carbohydrates, and protein into volatile fatty acids and thereby meet the majority of energy requirements for the body (6). Normal intestinal microbiota can stimulate the host immune system by improving intestinal self-recognition and immune ability of different bacteria. It has been well proven that intestinal microbiota play crucial roles in host physiology, health, and immune system maturation (7, 8). Any disruption of gut microbiota balance could contribute to host disorders, such as diarrhea, weakness, and immunosuppression (9, 10). Although gut microbiota communities associated with diarrheal livestock have been widely studied, especially in neonatal animals, analyses regarding the relationship between gut microbiota communities and adult yak have been insufficient to date.

Diarrhea poses a significant threat to animal husbandry development worldwide, which causes a serious disorder affecting fertility, milk production, and weight gain as well as leading to death in ruminants (11–13). Furthermore, clinical experience showed that chronic diarrhea in cattle is frequently encountered, due to the harsh cultural environment and lack of corresponding supervision, yaks frequently suffer from disease and typically display a higher incidence of bacterial diarrhea (9, 14). A full understanding of the gut microbiota of diarrheal yak is essential for further understanding the mechanisms causing ill and developing appreciative strategies to minimize the collateral damage. Metagenomics based on high-throughput sequencing has made it easier and faster than before to characterize gut microbial composition and diversity differences after suffering certain diseases. Moreover, there have been some recent publications that employed a high-throughput sequencing technique to study the gut microbiota of sheep, pigs, cows, and other animals suffering from diarrhea and identified microbial populations related to this disease (15–17). However, to date, there is less information available on the gut microbiota composition and diversity of diarrheic adult yaks. Therefore, the objectives of this study were to use 16S rRNA gene V3–V4 region sequencing to investigate the composition and variability of gut bacterial communities in the healthy and diarrheal adult yaks.

## Materials and methods

### Animals and sample collection

The experimental animals for this study were taken from adult domestic yaks (2–3 years old) in the Ganzi Tibetan Autonomous Prefecture, located in western Sichuan Province, China (approximately 3,700 m above sea level). All animals within a herd grazed night and day and were offered *ad libitum* access to water. Yaks were managed by staff trained to identify medical problems and diarrhea, and those with watery diarrhea, dehydration, and reduced feed and water intake were annotated and kept in a separate room. Only those with the symptoms lasting at least 2 days were defined as the occurrence of diarrhea, and fresh feces samples were collected using a sterile cotton swab from diarrheic yaks (case) on day 2 from the disease onset. At the same time, an age-matched healthy control yak (control) was sampled. Finally, from March to August 2021, a total of 30 fresh feces were obtained in the herd and then divided into two groups of the case (*N* = 15) and control (*N* = 15), respectively. All samples were deposited in 50 ml sterilized plastic tubes, transported to the laboratory, and then stored at −80°C until further evaluation.

### DNA extraction and sequencing

The microbial DNA was extracted from fecal samples using a QIAamp DNA Stool Mini Kit (Qiagen, Shanghai, China) based on the manufacturer's instructions. The concentration and purity were checked by Nanodrop ND1000 Spectrophotometer (Nanodrop Technologies, Montchanin, DE, USA) and gel electrophoresis, respectively. The V3–V4 regions of 16S rRNA genes were amplified using a specific primer (338F: 5′-ACTCCTACGGGAGGCAGCA-3′ and 806R:5′-GGACTACHVGGGTWTCTAAT-3′) with a barcode. The PCR protocol involved an initial denaturation step at 95°C for 3 min, 25 cycles of denaturation at 95°C for 30 s, annealing at 56°C for 30 s, extension at 72°C for 60 s, with a final extension at 72°C for 10 min. Each sample was amplified in triplicates to guarantee the accuracy of the results, and the amplicons were pooled and purified using an EasyPure PCR Purification Kit (TransGen, Beijing, China). Qualified amplicons were used to produce sequencing libraries using Illumina TruSeq (Illumina, San Diego, CA, USA) following the manufacturer's specifications. Finally, the libraries were diluted and mixed in proportion and sequenced on Illumina HiSeq 2500 platform to generate 250 bp paired-end reads.

### Bioinformatics and data analysis

The raw reads were assigned to samples based on their unique barcode, and the Trimmomatic (v0.33) (18) and fastp software (v0.19.8) (19) were used to screen the qualified raw reads. Cutadapt software (1.9.1) (20) was enrolled to identify and trim the adaptor sequences for obtaining high-quality target reads. Sequence analysis was processed using the open source software Qiime2 (21) for paired-end reads' merging, demultiplexing, and *de novo* operational taxonomic unit (OTU) picking. Representative OTU sequences were aligned using the DEBLUR program (22) integrated within QIIME2. A Naïve Bayesian classifier was trained on the Silva reference sequences (138 clustered at 99% similarity) and used to classify these OTUs into specific taxa. For each sample, the community richness (Chao1 and observed features) and diversity (Shannon and Simpson indices) were calculated using QIIME2 by the Kruskal-Wallis test (21, 23). Beta diversity was calculated through the Bray-Curtis, Jaccard, Weighted UniFrac, and Unweighted UniFrac metric using permutational multivariate analysis of variance (PERMANOVA) method to evaluate the dissimilarity and distance between the animals of the same group with the QIIME2 platform (21, 23). Dissimilarities in fecal bacteria were visualized using principal coordinates analysis (PCoA) method. Moreover, the rarefaction and rank curves were generated to assess the sequencing depth, richness, and evenness. Finally, the linear discriminant analysis (LDA) effect size (LEfSe) was conducted to assess whether an important microbiome resulted in differences (24), where a score >4 was considered as an important contributor to the model. Statistical analyses were performed using R (v4.1.3) software (https://www.r-project.org/). The criterion of significance was conducted at *p* < 0.05, and the values were presented as the means.

### Data availability

All 16S rRNA gene sequencing data in this study can be freely retrieved from the ENA database (https://www.ebi.ac.uk/ena/browser/) with study accession no. ERP137909.

## Results

### Analysis of sequencing data and taxonomy

A total of 2,398,491 pair-end reads with an average of 79,950 per sample were generated using Illumina HiSeq 2500 platform. After quality control processing and eliminating the unqualified data, a total of 2,363,058 high-quality reads were obtained from all the samples, with an average of 78,769 reads per sample (ranging: 78,301–79,218). The rarefaction curves indicated that with the deepening test depth, their slopes gradually decreased and showed a saturated tendency when the number of qualified sequences was more than 5,000, this finding indicated that the sequencing quantity and depth met the requirement for subsequent analysis (Supplementary Figure S1). According to DEBLUR program, a total of 3,904 OTUs were identified based on clustering at the single-nucleotide level, and these OTUs were assigned to 12 bacterial phyla, and more than 80% of the constructed OTUs were taxonomically assigned to 198 genera. Among these taxonomically OTUs, phyla Bacteroidota and Firmicutes were absolutely predominated with the observed frequencies of 56.3 and 27.9%, respectively.

### Alterations in gut microbial diversities with the effect of diarrhea

**Figure 1:**
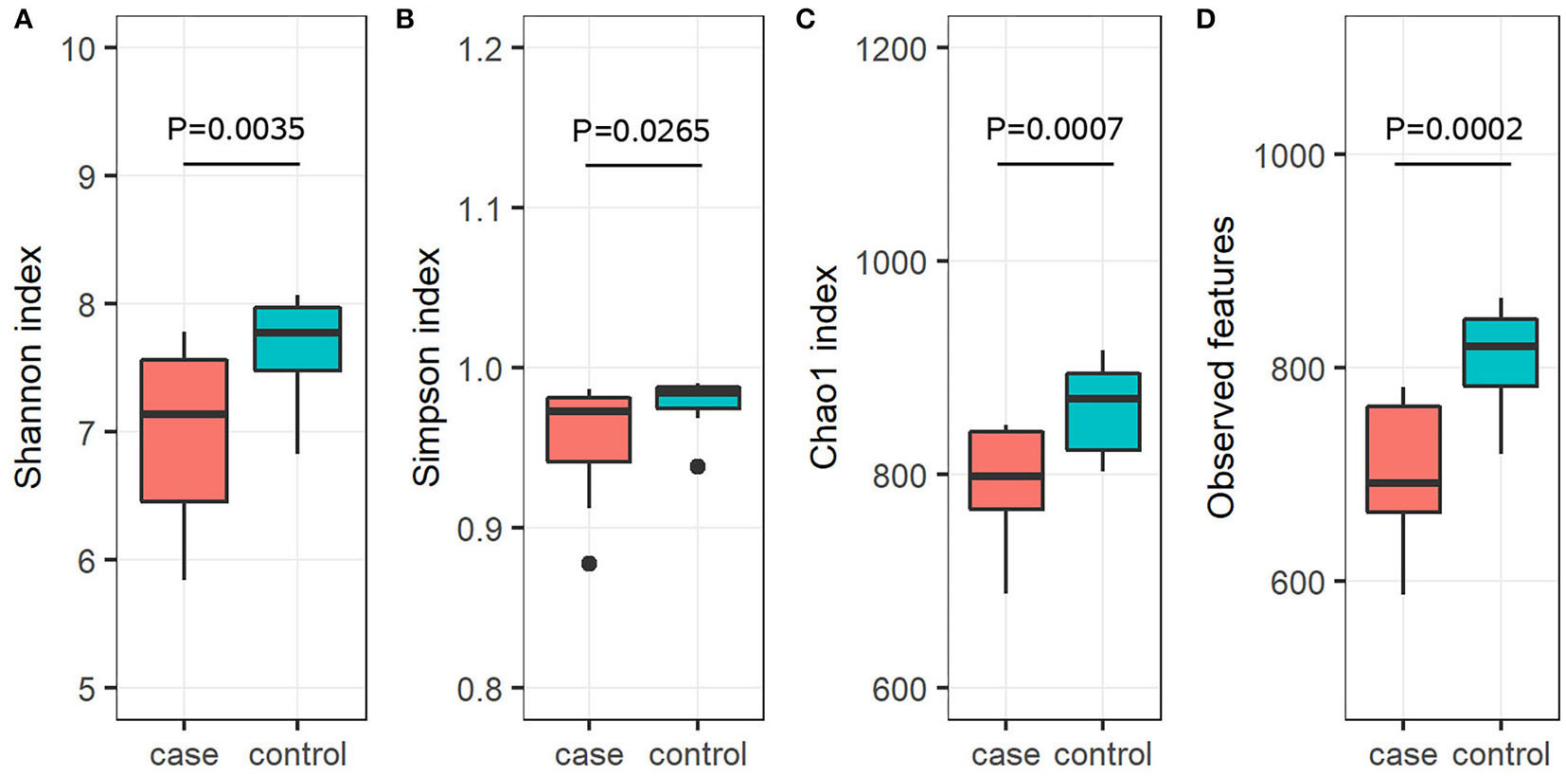
Gut microbial alpha diversity analysis. Diversity in the gut microbiota community was measured using the Shannon index **(A)**, the Simpson index **(B)**, the Chao1 index **(C)**, and observed features **(D)**. The bottom and top of each box are the first and third quartiles, respectively, and the band inside the box is the median.

**Figure 2:**
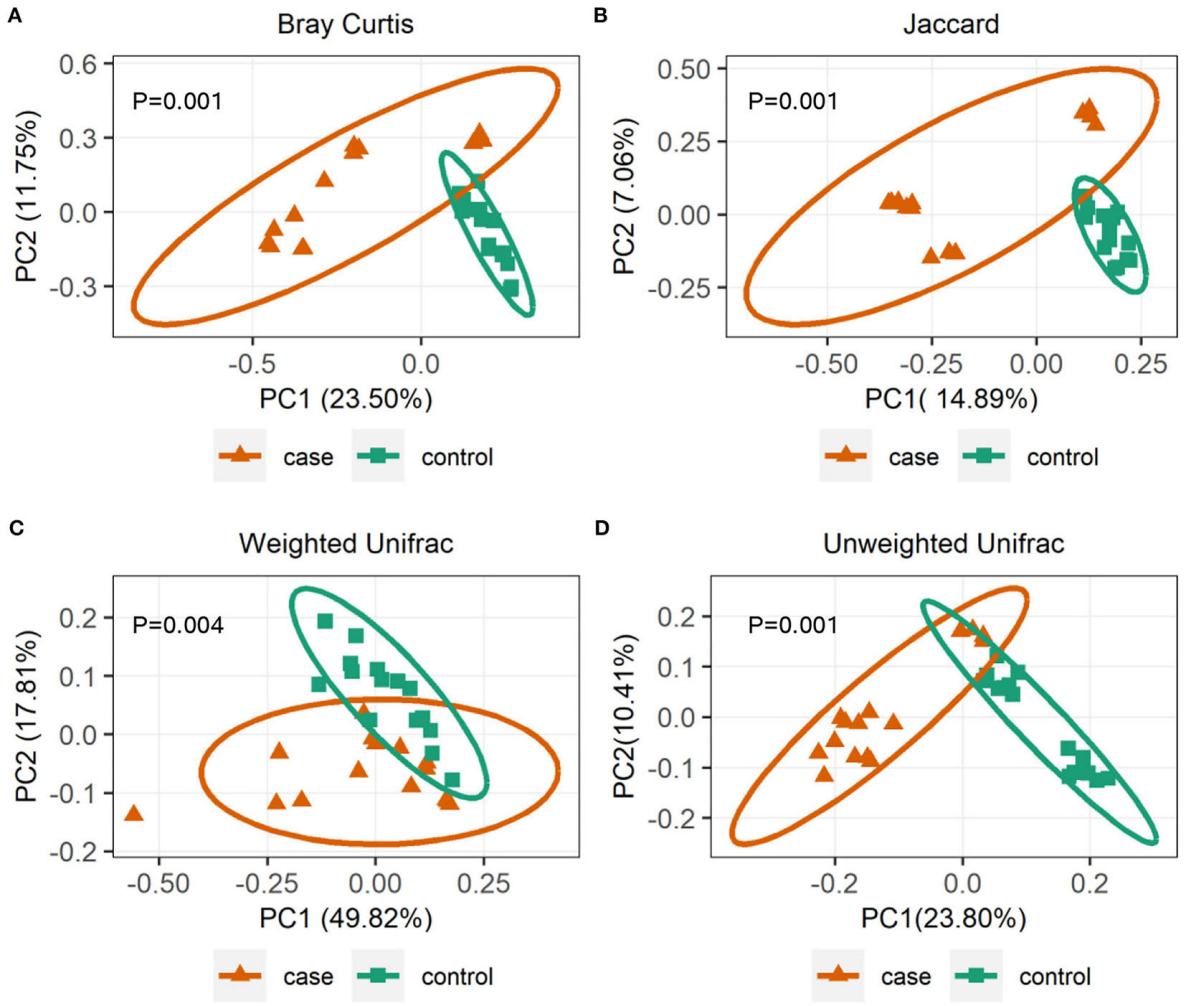
Gut microbial beta diversities analysis. Principal coordinate analysis (PCoA) plots based on community membership as measured by the Bray-Curtis distances **(A)**, Jaccard distances **(B)**, Weighted UniFrac distances **(C)**, and Unweighted UniFrac distances **(D)**. Orange triangles and blue squares circle represent case and control yaks, respectively.

To further investigate the alpha diversity of gut microbiome in both grouped animals, the species diversity (Shannon and Simpson indices) and species richness (Chao1 index and observed features) were analyzed. From the point of view of microflora abundance, the Shannon and Simpson indices were 6.81 and 0.94, and 7.67 and 0.98 for the case and control groups, respectively, and revealed that the case group had significantly lower gut microbial abundance (*p* < 0.05; Figures 1A,B). In terms of flora diversity, the Chao1 index and observed features were 780.92 and 689.47, and 864.65 and 810.13 for the case and control groups, respectively, revealing significantly lower indices of the case group when compared to the control group (*p* < 0.01; Figures 1C,D). These results indicated that diarrhea significantly decreased the gut microbial abundance and diversity of yaks. To assess the dissimilarities in community structure and membership of gut microbiome between groups, beta diversity metrics (Bray-Curtis, Jaccard, Weighted UniFrac, and Unweighted UniFrac) were calculated. The PCoA plots based on those metrics showed that individuals in the control group were clustered together and significantly separated from the case group (all *p* < 0.05; Figures 2A–D), indicating that there were great differences between the case group and the control group.

### Difference in gut microbiota composition in the control and case yaks

**Figure 3:**
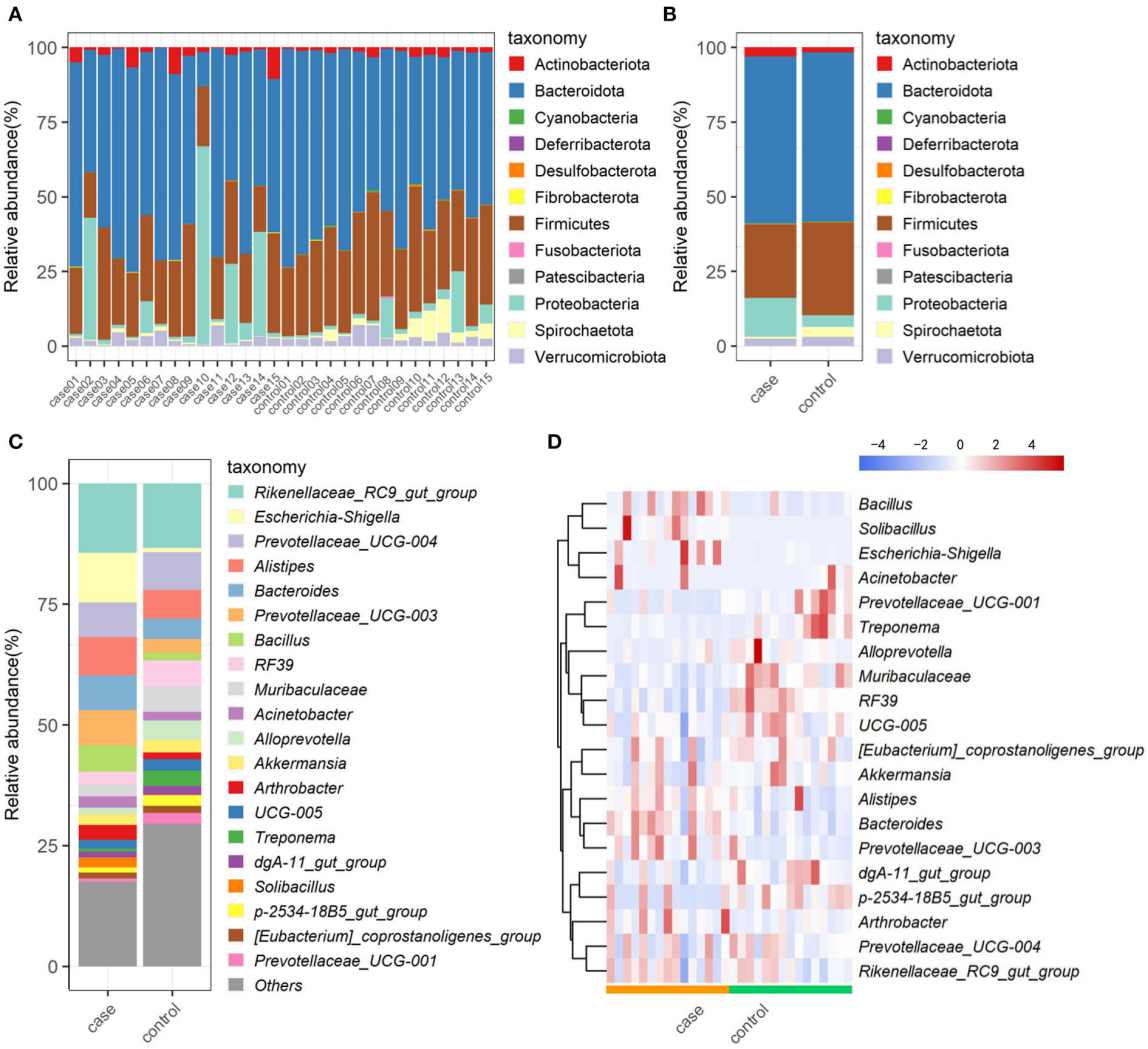
The composition and relative abundance of gut microbiota. Multicolored stacked bar graphs represent the relative abundance of each bacterial taxon assignment at the phylum level in each sample **(A)** and both groups **(B)**; taxon assignments at the genus level (top 20) in both groups **(C)**; hierarchically clustered heatmap of taxonomy analysis at the genus in each sample **(D)**.

The relative proportions of preponderant taxa at levels of phylum and genus were calculated in all samples, from which we observed considerable variability between case and control animals. As shown in Figure 3A, a total of 12 phyla are identified from the 30 samples. According to the classification results, Bacteroidota (56.28%), Firmicutes (27.87%), and Proteobacteria (8.44%) were the three most dominant in all samples, which accounted for approximately 93% of the taxonomic groups identified. Other phyla, such as Verrucomicrobiota, Actinobacteriota, Spirochaetota, Cyanobacteria, Desulfobacterota, Patescibacteria, Fibrobacterota, Fusobacteriota, and Deferribacterota, presented a lower abundance. Interestingly, Proteobacteria in the case group was much more abundant than that in the control group, whereas, Firmicutes and Spirochaetota were decreased in the case group as compared to the control group (Figure 3B). At the genus level, the most abundant taxonomy was *Rikenellaceae_RC9_gut_group* (13.55%), followed by *Prevotellaceae_UCG-004* (7.91%), and *Alistipes* (5.87%) in the control group. Especially, an obvious difference in predominant bacterial genera was observed in the case group, in which, *Escherichia-Shigella* (15.08%), *Rikenellaceae_RC9_gut_group* (13.34%), and *Alistipes* (7.56%) were the most abundant taxonomy (Figure 3C). The relative abundances of genus *Escherichia-Shigella* and *Prevotellaceae_UCG-003* were dramatically increased, whereas *Treponema, p-2534-18B5_gut_group*, and *Prevotellaceae_UCG-001* were observably decreased with the effect of diarrhea. The abundance alterations of these bacteria could be the primary reason for the diarrhea of yak. Moreover, the distribution of bacterial genera in each sample could also be observed in the heatmap (Figure 3D).

**Figure 4:**
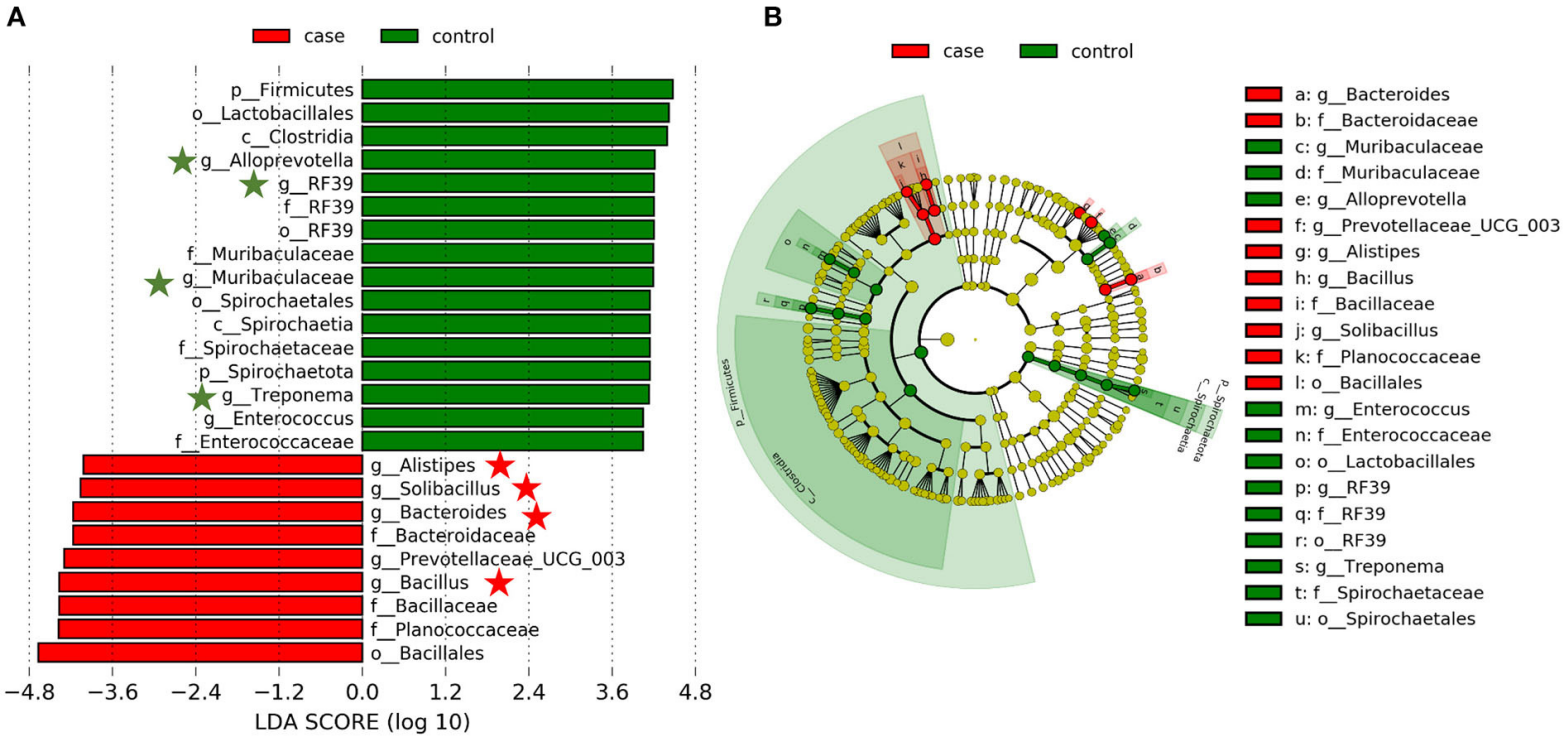
Linear discriminant analysis effect size (LEfSe) analysis and linear discriminant analysis (LDA) characterized the gut microbiota. **(A)** LDA scores indicated differences in abundance between the case and control groups (LDA scores > 4.0). **(B)** Cladogram using LEfSe method revealed the phylogenetic distribution of gut bacterial community associated with the case (red) and control (green) groups.

Based on our results, we have found that the gut microbiome of case and control animals was largely altered, but this discriminant analysis cannot distinguish the primary taxon, we further detected the specific bacteria associated with diarrhea using LEfSe analysis. As shown in Figure 4, a total of 16 and 9 bacterial taxa are abundant in the control and case groups, respectively. At the genus level, *Alistipes, Solibacillus, Bacteroides, Prevotellaceae_UCG_003*, and *Bacillus* were significantly enriched in the case group, while *Alloprevotella, RF39, Muribaculaceae, Treponema*, and *Enterococcus* were mostly associated with health group based on LDA method. Interestingly, eight of these genera (marked by five-pointed stars) were listed in the top 20 abundant taxa (Figure 4A). Furthermore, a cladogram representing the taxonomic hierarchical structure of gut microbiota indicated a significant difference in phylogenetic distributions between the case and control groups (Figure 4B). These results showed a remarkable difference in gut microbiota composition between diarrheic and healthy yaks.

## Discussion

Intestinal diarrhea is a widely prevalent disease in the livestock industry, which is deemed as a crucial factor resulting in the reduction of production, and causes approximately half of all deaths in ruminants (12, 25). Previous studies showed that there are umpteen potential causative factors of diarrhea in bovines, such as pathogenic agents, weaning, management factors, and nutritional, physiological, and environmental stresses (26, 27), most of these factors have been linked to the imbalances of normal intestinal flora as they play an important role in animal's intestinal function. A number of recent studies have utilized microbiome analysis to characterize the gut microbiota of diarrhea in domestic livestock, especially focused on lambs (28), commercial piglets (15), early-weaned Tibetan piglets (29, 30), neonatal dairy calves (31, 32), and sucking goats (33). Furthermore, these studies have provided evidence that gut microbial dysbiosis might have mechanistic relevance to diarrhea. Han et al. (9) investigated the differences in the intestinal microbiome of diarrheic and healthy perinatal yaks using a high-throughput sequencing technique and found significant differences in the number and structure of intestinal flora. A previous study showed that the main bacterial phyla in the intestinal tissues of yak were Firmicutes (36.8 ± 14.2%), Bacteroidetes (29.4 ± 10.5%), and Proteobacteria (24.9 ± 10.9%) (9). However, no studies have evaluated the alterations of gut microbiota of adult yaks suffered from diarrhea. The present study investigated the composition and variation of gut microbial communities of adult yaks with or without diarrhea. To the best of our knowledge, this was the first report using a high-throughput sequencing approach to comprehensively explore the gut microbiota composition and diversity between the healthy and diarrheic adult yaks.

Generally, the animal gut microbiota is not fixed but can be affected by many factors, such as genetics, age, sex, diet, and health status (34). Diarrhea poses a significant threat to the livestock industry, which can result in a significant decrease in the diversity of gut microbiota and will affect intestinal function (35). There has been reported significantly decreased alpha diversity of gut bacterial community on diarrheic rats (36) and piglets (37). Consistent with those publications, this study demonstrated a significantly lower alpha diversity of diarrheic yaks (Figures 1A–D), implying the gut microbiome dysbiosis. However, not in line with the previous report describing the alpha diversity of gut microorganisms in diarrheal perinatal yaks (9), these differences may be attributed to diarrhea *per se* but also be associated with the substantial difference in development phases (38). To further infer bacterial community diversity between samples, PCoA analyses using Bray-Curtis, Jaccard, Weighted UniFrac, and Unweighted UniFrac metrics were performed. It was interesting to find that the control samples were obviously clustered together and significantly separated from case samples (Figures 2A–D), indicating that the gut bacterial community had the same trend in health yaks as compared to the diarrheic animals.

In this study, a total of 12 phyla were successfully identified, of which, Firmicutes, Proteobacteria, and Bacteroidetes were the most abundant phyla in yaks regardless of the health status, which is consistent with previous publications on other ruminants (16, 31), indicating their key roles in intestinal function. Along with the occurance of diarrhea, the abundance of Firmicutes decreased, whereas that of Proteobacteria significantly increased (Figures 3A,B). In bovine, Firmicutes is mainly responsible for decomposing fiber and cellulose, so less abundance of which may affect the host's energy and nutritional demands (39). Furthermore, most members of Firmicutes could contribute to improving the intestinal environment and against pathogenic invasion (40). As for Proteobacteria, there are many opportunistic pathogens and pathogenic bacteria members, whose abundant increase may be one of the causes of this disease (41). At the genus level, the relative abundances of *Escherichia-Shigella* were dramatically increased with the effect of diarrhea. It is well known that *Escherichia-Shigella* is highly related to diarrhea (42), which may illustrate the importance of this bacterium as a cause of diarrhea in yaks. Furthermore, LEfSe results showed that *Alistipes, Solibacillus, Bacteroides, Prevotellaceae_UCG_003*, and *Bacillus* genera were significantly enriched in the case group, and four of them were listed in the top 20 abundant taxa (Figure 4A). These results revealed that these bacteria genera played important roles in diarrheal yaks.

There were a few noteworthy limitations to this study. First, a relatively small sample size (15 cases and 15 controls) was conducted to explore the gut microbiota profiles of diarrheal yaks, which might contribute to problems of reproducibility, i.e., false positives and false negatives. Second, the fecal samples were collected instead of the gut; there may be some limitations in exploring the gut microbiota of diarrheal yaks. However, fecal samples might be valuable sample sources for investigating diseases and biomarkers in humans and animals, which can provide reliable information about the host (43). Variation in gut microbiome composition was found to dominate differences between individuals instead of collection-processing methods or day of collection (44). Third, 16S rRNA gene sequencing is a powerful tool for understanding the linkage between the microbial community and disease, but a significant challenge is discriminating cause-and-effect relationships (44, 45), and results of which are relative rather than absolute since the taxonomy assignment is reliant on the completeness of reference databases, such that the actual quantity of a particular bacterium is uncertain. Together, the results of this study could help broaden our understanding of diarrhea in adult yaks without inferring the main pathogenic bacteria.

## Conclusion

In summary, the present study herein demonstrated the significant alterations of gut microbial composition and structure in diarrheal adult yaks and is characterized by decreased gut bacterial alpha diversities and altered gut bacterial compositions. These results may help broaden our understanding of diarrhea for developing an effective treatment strategy for this disease.

## Data availability statement

The datasets presented in this study can be found in online repositories. The names of the repository/repositories and accession number(s) can be found below: European Nucleotide Archive PRJEB53132.

## Ethics statement

The animal study was reviewed and all experimental procedures involved in this study were approved by the Institutional Animal Care and Use Committee of Chengdu University (SSXY-600008).

## Author contributions

Z-LW, WW, and XT: conceived and designed the experiments. Z-LW, XT, DY, and RW: performed the experiments. Z-LW and RW: analyzed the data. Z-LW: wrote the paper. JZ, DL, and WW: reviewed and edited the manuscript. All authors read and approved the final version of the manuscript.

## Funding

This work was supported by the Youths Fund of Natural Science Foundation in Sichuan Province (No. 2022NSFSC1746), the Foundation for Young PhD Teachers of Chengdu University (No. 2081921068), the Opening Foundation of Meat Processing Key Laboratory of Sichuan Province (No. 21-R-36), and the Sichuan Science and Technology Program (No. 2020YFN0153).

## Conflict of interest

The authors declare that the research was conducted in the absence of any commercial or financial relationships that could be construed as a potential conflict of interest.

## Publisher's note

All claims expressed in this article are solely those of the authors and do not necessarily represent those of their affiliated organizations, or those of the publisher, the editors and the reviewers. Any product that may be evaluated in this article, or claim that may be made by its manufacturer, is not guaranteed or endorsed by the publisher.
