# Supplementary material for: Characterization of gut microbiota dysbiosis of diarrheic adult yaks through 16S rRNA gene sequences
Source: Front Vet Sci. 2022 Sep 9;9:946906. doi: 10.3389/fvets.2022.946906 (PMC9500532; doi:10.3389/fvets.2022.946906)
Supplement: Supplementary file 1 [file Data_Sheet_1.docx]

## Supplementary Figures


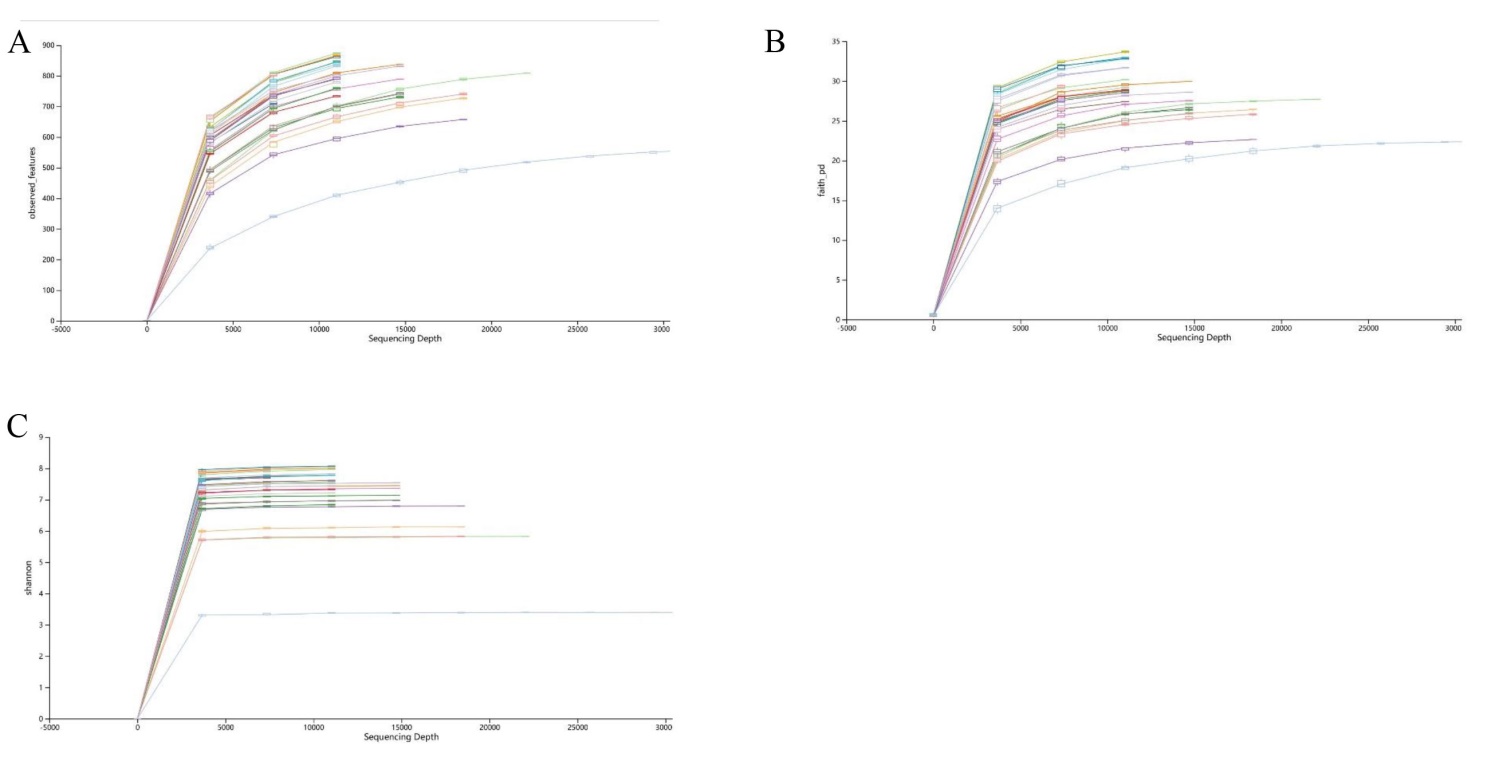


**Figure S1. Rarefaction curves for the gut microbiota.** Curves of observed features (A), faith PD (B), and shannon indices (C).
